# Draft genome of *Ompok bimaculatus* (Pabda fish)

**DOI:** 10.1186/s13104-019-4867-y

**Published:** 2019-12-26

**Authors:** Ruby Dhar, Karthikeyan Pethusamy, Sunil Singh, Indrani Mukherjee, Ashikh Seethy, Bharoti Sengupta, Tryambak Srivastava, Sajib Sarkar, Vikramjit Mandal, Madhumita Karmakar, Sandipan Gupta, Arpita Ghosh, Subhradip Karmakar

**Affiliations:** 1 Department of Biochemistry, All India Institute of Medical Sciences, New Delhi, India; 2 Eurofins Genomics India Pvt Ltd, Bangalore, India; 3 Department of Industrial Fish and Fisheries, Bramhananda Keshab Chandra College, West Bengal State University, Kolkata, India

**Keywords:** Butter catfish, *Ompok bimaculatus*, Siluridae, Whole genome sequence, NGS

## Abstract

**Objective:**

Pabda (*Ompok bimaculatus*) is a freshwater catfish, largely available in Asian countries, especially in Bangladesh, India, Pakistan and Nepal. This fish is highly valued for its fabulous taste and high nutritional value and is very popular as a rich source of proteins, omega-3 and omega-6 fatty acids, vitamins and mineral for growing children, pregnant females and elders. We performed *de*-*novo* sequencing of *Ompok bimaculatus* using a hybrid approach and present here a draft assembly for this species for the first time.

**Data Description:**

The genome of *Ompok bimaculatus* (Fig. 1: Table 1, Data file 3) from Ganges river, has been sequenced by hybrid approach using Illumina short reads and PacBio long reads followed by structural annotations. The draft genome assembly was found to be 718 Mb with N50 size of 81 kb. MAKER gene annotation tool predicted 21,371 genes.

## Objective

Pabda fish is a freshwater catfish, with its fabulous taste and high nutritional value. *Ompok bimaculatus* (Family: Siluridae), also known as Indian butter catfish or commonly known as pabda, has fascinated considerable attention in diversification due to its good taste, high nutritional value and soft bony structure [1–3]. Pabda is largely available in Asian countries, especially in Bangladesh, India, Pakistan and Nepal. Catfishes are an excellent source of omega-3 and omega-6 fatty acids, vitamins and mineral those are excellent for growing children, pregnant females and elders. Overexploitation for food is a major threat and has resulted in remarkable population decline [4, 5]. The fish is in declining phase due to lack of definite information on the biological aspects accompanied by declining population owing to habitat loss, indiscriminate pesticide and weedicide use, loss of breeding grounds and overfishing valued for its unique taste has pushed the population of Pabda in IUCN red list of threatened species [3, 5].

Knowing the complete genome of this fish will help in better understanding of the genome organization, evolution as well as for conservation and farming applications [6]. This involves steeping up of breeding process, as well as to identify lineage specific changes that are critical for its adaptation besides knowing about the risk factors as well as its immune system that helps in its survival in the wild or in captivity.

## Data description

Fresh *Ompok bimaculatus* which were approximately 4 months old were freshly caught from the Ganges river in India and instantly used for DNA extraction. The taxonomic identification of this fish has been confirmed following standard taxonomic keys like studying the fin formula and other standard morphological characteristics. Muscle tissue was dissected from this fish and high-molecular weight genomic DNAs was purified from one specimen using Qiagen Genomic-tip 100/G as per the manufacturer’s instruction. The quality and quantity of the isolated genomic DNA was checked on NanoDrop followed by 0.8% Agarose gel.

Two pair-end libraries were constructed for Illumina sequencing and one SMRTbell library was constructed for Pacific Bioscience Sequel, single molecule, real time (SMRT, Single Molecule Real Time) sequencing platforms as mentioned in Table 1, Date file 1. The quality of the reads was checked using FastQC (Table 1) [7].

**Table 1:** Overview of data files/data sets

Label	Name of data file/data set	File types (file extension)	Data repository and Identifier (DOI or accession number)
Data file 1 [16]	Sequencing statistics	Table	10.6084/m9.figshare.9827312.v1
Data file 2 [17]	Genome assembly statistics	Table	10.6084/m9.figshare.9830954.v1
Data file 3 [18]	Fish Pictures	Figure 1	10.6084/m9.figshare.9831155.v1
Data file 4 [19]	Whole genome assembly data	FASTA	https://www.ncbi.nlm.nih.gov/bioproject/PRJNA552450
Data file 5 [20]	Whole genome sequence	FASTA	https://www.ncbi.nlm.nih.gov/assembly/GCA_009108245.1

MaSuRCA (Maryland Super- Read Celera Assembler) v3.2.8 was used for hybrid de novo assembly [8] using both the Illumina and PacBio data. The genome assembly has been deposited in the NCBI GeneBank under the Bioproject ID: PRJNA552450 (Table 1, Data files 2, 4). The assembled genome size of *Ompok bimaculatus* is 718 Mb and approximately 72% of the genome has been assembled as per the in silico genome size estimation.

The BUSCO v3 [9] analysis revealing 85.7% completeness, indicating the genome to be of good quality. MAKER v3.0 pipeline [10] was used for structural annotation. GC content of the genome was determined to be 38.84%. RepeatMasker v4.0.9 was used with the latest version of Repbase database [11, 12], repeat elements identified were 7.87%. Altogether, 21,371 genes were predicted by the MAKER gene annotation pipeline using proteins from channel catfish. Out of the 21,371 genes, 20,923 were annotated using Diamond [13] (BlastX mode) against NCBI ‘NR’ database and 5589 genes were found to have GO (Gene Ontology) term assigned to them. The butter catfish genome was found to be comparable to *Ictalurus punctatus* (Channel catfish, 892 Mb genome and 27,156 genes) [14] and to the genome of *Pangasianodon hypophthalmus* (Striped catfish, 715 Mb genome and 24,083 genes) [15].

## Limitations

The number of the scaffolds containing N in the genome are 27 and a total of 3773 bases are positioned in this gap region. The assembled genome size of the Indian butter catfish is 718 MB as compared to in silico estimated genome size of 992 Mb.

## Data Availability

The data described in this Data note can be freely and openly accessed via figshare, Refer Table 1 for details and links. The genome assembly has been deposited in the NCBI GeneBank under the Bioproject ID: PRJNA552450 (Table 3).

## Abbreviations

*BUSCO*: Benchmarking Universal Single-Copy Orthologs
*MaSuRCA*: Maryland Super-Read Celera Assembler
*IUCN*: International Union for conservation of nature
*PacBio*: Pacific Bioscience
*GO*: Gene Ontology
*SMRT*: single molecule real time sequencing
